# Hidden Solid-State Transformation of Darunavir in Low-Temperature Hot-Melt-Extruded Granules: Implications for Pharmacy Compounding and Routine Quality Control

**DOI:** 10.3390/pharmaceutics18091079

**Published:** 2026-08-27

**Authors:** Mark Mandrik, Veronika Makarova, Ludmila Korol, Ivan Sadkovskii, Ivan Krasnyuk, Sergey Antonov

**Affiliations:** 1 A.P. Nelyubin Institute of Pharmacy, Sechenov First Moscow State Medical University, 8-2 Trubetskaya str., 119991 Moscow, Russia; 2 A.V. Topchiev Institute of Petrochemical Synthesis, Russian Academy of Sciences, Leninsky pr. 29, 119991 Moscow, Russia

**Keywords:** hot-melt extrusion, pharmacy compounding quality control, personalized medicines, darunavir, solid-state stability, X-ray diffraction, differential scanning calorimetry, polymeric premix

## Abstract

**Background:** Hot-melt extrusion (HME) is a scalable pharmaceutical technology increasingly relevant to flexible manufacturing, including small-batch production, personalized dosage-form development, and potential use in pharmacy compounding. When translated into compounding practice, however, HME introduces a risk that routine quality-control methods available in pharmacies may be insufficient to reliably assess the stability of extrusion-based preparations. **Methods:** Granules containing 50% (*w*/*w*) darunavir were prepared by HME at 70 and 90 °C using a previously developed polymeric premix. Samples were stored for 24 months under ambient conditions. During storage, routine quality attributes were evaluated, including appearance, particle size distribution, loss on drying, disintegration time, content uniformity, and assay. Solid-state changes were investigated using differential scanning calorimetry (DSC) and X-ray diffraction (XRD), with a reference PEG-associated darunavir sample prepared and characterized for comparative analysis. Changes in drug release and darunavir content were assessed by dissolution testing and HPLC analysis, respectively. **Results:** Granules produced at both extrusion temperatures retained acceptable routine quality attributes throughout the 24-month storage period. No substantial changes were detected by visual inspection, pharmacopoeial tests, or UV assay. However, DSC revealed a new thermal event after storage, while XRD showed the formation of a new crystalline phase. Comparison with the reference PEG-associated sample supported the assignment of this phase as a PEG-associated crystalline phase of darunavir. Importantly, this transformation occurred even though the routine quality attributes evaluated in pharmacy compounding practice remained unchanged. Dissolution profiles differed between samples tested immediately after preparation and after long-term storage, with a more pronounced overall difference for granules produced at 90 °C, whereas HPLC confirmed comparable darunavir content in all investigated samples. **Discussion:** Our results show that routine compounding quality control can meet conventional acceptance criteria while failing to detect API solid-state changes in the investigated HME-derived system. In the PEG-containing matrix, amorphous darunavir undergoes storage-induced crystallization, forming a PEG-associated crystalline phase consistent with its known affinity for polyol-containing media. **Conclusions:** Acceptable routine quality attributes do not necessarily reflect the solid-state stability of APIs in HME-based formulations. These results highlight the need for solid-state risk assessment when developing extrusion-based systems intended for pharmacy compounding and other personalized manufacturing models in which routine quality control may not include advanced solid-state characterization.

## 1. Introduction

Hot-melt extrusion (HME) is an established pharmaceutical technology platform with broad application potential [1]. Although HME has traditionally been valued for its ability to amorphize active pharmaceutical ingredients (APIs) and enhance the solubility of poorly water-soluble compounds, its relevance is increasingly shifting toward flexible pharmaceutical manufacturing models [2,3]. This shift is particularly relevant in the context of personalized pharmacotherapy, where technologies are required not only to provide scalability but also to facilitate the adaptation of dosage forms to specific clinical needs [4].

A clear example of this trend can be found in fused deposition modeling (FDM), where HME is used to produce API-loaded filaments [5,6]. These filaments can subsequently be employed for the 3D printing of dosage forms with predefined doses and geometries [7,8,9,10]. In addition, conventional granules or pellets may also be produced from such filaments. Consequently, HME serves as a technological link between industrial pharmaceutical manufacturing and personalized pharmacotherapy, including flexible small-batch production and potential adaptation to pharmacy compounding [11,12].

Within this manufacturing paradigm, standardized intermediate products become particularly important [13]. One such example is an API-free polymeric premix, which represents a pre-engineered matrix with predefined physicomechanical properties suitable for HME processing [14]. From a practical perspective, the use of premixes can substantially simplify pharmaceutical development [15]. For pharmacy compounding, this approach is conceptually attractive because a standardized intermediate product could serve as a platform to which an API is added to obtain the required dosage.

At the same time, the implementation of HME in flexible manufacturing should not be viewed simply as a reduction in production scale. In industrial small-batch manufacturing, reduced batch size does not imply reduced analytical control, since such products remain subject to conventional pharmaceutical development, validated analytical methods, and appropriate quality-control strategies [16,17]. A more specific concern arises when HME-based systems are translated into pharmacy-compounding practice.

Accordingly, quality control in pharmacy compounding is largely risk-based and process-oriented, with final product testing serving as a selective rather than universal safeguard and with an analytical scope that differs substantially from that typically applied in industrial pharmaceutical manufacturing [18,19,20]. Its robustness therefore depends on the maturity of the compounding process, personnel practices, documentation, and the justification of beyond-use dating within a particular facility. In the case of solid non-sterile compounded dosage forms, routine quality-control procedures available in pharmacy practice are generally limited to basic pharmacopoeial and procedural attributes, such as visual appearance, dosage accuracy, mass uniformity, packaging, labelling, review of compounding records, and related documents [21]. As a result, changes in the solid-state properties of active pharmaceutical ingredients may remain outside the analytical scope of routine compounding quality control unless additional characterization is specifically performed [22].

Therefore, a key question is whether the quality and stability of HME-produced dosage forms can be reliably assessed using routine quality-control methods available in pharmacy practice. This issue is particularly relevant for amorphous solid dispersions. Although the amorphization of APIs can enhance dissolution and potentially improve the biopharmaceutical performance of poorly soluble compounds, the amorphous state is thermodynamically unstable [23]. Its stability depends on molecular mobility, moisture content, storage temperature, API–polymer interactions, and the intrinsic tendency of the API to recrystallize [24,25]. Consequently, the physical stability of the amorphous phase represents a critical quality attribute [26].

An even more challenging situation arises when APIs exhibit a tendency toward solvatomorphic crystallization [27,28]. In such cases, solid-state evolution depends on the interplay between the molecule’s intrinsic tendency toward structural ordering and the chemical nature of its surrounding environment [29]. Polymer matrices containing low-molecular-weight plasticizers can direct the pathway of API phase transformation [30,31,32]. As a result, a dosage form that appears physically stable according to routine compounding quality-control procedures may gradually transition into a new solid-state form that cannot be detected without specialized analytical techniques [33].

Darunavir represents a particularly relevant model compound in this context. It is a poorly water-soluble API known to form solvated crystalline phases [34,35]. According to the Merck Index, darunavir is reported as an amorphous solid with a melting point of approximately 74 °C accompanied by decomposition, making it a suitable model compound for assessing the effects of low-temperature thermal processing [36,37,38,39]. Consequently, darunavir offers an opportunity to investigate extrusion under conditions approaching its thermal stability limits. At the same time, its solvatomorphic behavior makes it suitable for assessing whether routine quality-control procedures available in pharmacy practice can reveal storage-induced solid-state transformations [17].

Of particular importance to the present study is the interaction of darunavir with polyol-containing media. In our previous work, laser microinterferometry demonstrated that darunavir is practically insoluble in water and oils but exhibits good solubility in several alcohols and glycols, especially polyethylene glycol 400 (PEG 400), a plasticizer widely used in pharmaceutical extrusion. Notably, the darunavir–PEG 400 system showed the gradual formation of crystalline solvated phases during storage [40]. These findings provide a mechanistic rationale for investigating whether incorporation of darunavir into a PEG-containing polymer matrix can promote directed solid-state transformation of the API during storage [41,42].

To test this hypothesis, darunavir-loaded granules were prepared by HME using a previously developed polymeric premix based on the transient plasticization window concept and designed for low-temperature extrusion processing [14]. The study was specifically structured to compare the results of routine quality-control procedures available in pharmacy compounding practice with data obtained by differential scanning calorimetry (DSC) and X-ray diffraction (XRD), which provide direct information on the solid-state characteristics of the API.

Accordingly, the objective of the present study was to evaluate whether routine quality-control parameters used in pharmacy compounding are sufficient to reveal solid-state changes in darunavir granules produced by hot-melt extrusion during long-term storage. In addition, the study examined whether darunavir could be processed using the previously developed polymeric premix at different extrusion temperatures and how these conditions affected extrusion performance and the API content of the resulting granules.

## 2. Materials and Methods

### 2.1. Materials

Amorphous darunavir (Henan Tianfu Chemical Co., Ltd., Zhengzhou, China) with a water content of 0.4% was used as the model API. Darunavir-loaded granules were prepared by hot-melt extrusion using the polymeric system as the carrier. The polymeric system had the following composition (wt.%): polyvinylpyrrolidone (PVP) K-29/32 (Plasdone K-29/32) (USP/EP-grade, Ashland Industries Europe GmbH, Schaffhausen, Switzerland)—32, polyethylene glycol 400 (PEG 400) (USP/EP-grade, Merck, Darmstadt, Germany)—22, polyethylene glycol 1500 (PEG 1500) (USP/EP-grade, Clariant AG, Muttenz, Switzerland)—19, hydroxypropylcellulose 80000 (HPC EF) (Klucel EF) (USP/EP-grade, Ashland Industries Europe GmbH, Schaffhausen, Switzerland)—27%.

Methanol (≥99.9%, HPLC-grade, Chimmed, Moscow, Russia), acetonitrile (≥99.9%, HPLC-grade, Cryochrom Ltd., Saint Petersburg, Russia), phosphate buffer solution, and purified water compliant with the European Pharmacopoeia were used for analytical and pharmacopoeial testing.

### 2.2. Preparation of Darunavir-Loaded Granules by Hot-Melt Extrusion

Darunavir-loaded granules were prepared by hot-melt extrusion using the polymeric system. Amorphous darunavir and the polymeric carrier were used at a mass ratio of 50:50. Granules of the polymeric premix and darunavir powder were co-fed into the extruder feed zone. The feed rate was adjusted only to maintain continuous feeding and was not optimized as an independent process variable.

Extrusion was performed using a co-rotating twin-screw extruder Scientific LTE16-36 FAC/00 (Labtech Engineering, Samut Prakan, Thailand) with a screw diameter of 16 mm, a barrel length of 32 L/D, and a die diameter of 2 mm. The extruder was equipped with eight independently controlled heating zones and an additional die heater. The screw configuration consisted of four conveying sections alternating with three mixing zones.

Granules were prepared in multiple runs under two processing conditions corresponding to melt temperatures of 70 and 90 °C. Extrusion was carried out under isothermal conditions, with the same temperature maintained across all barrel zones and the die. In all experiments, the maximum recorded melt temperature did not exceed the set values. The extrudate began to emerge approximately 5 min after feeding started, corresponding to the minimum residence time of the material in the extruder barrel.

The extrudate was pelletized immediately after exiting the die, prior to complete solidification, using a strand pelletizer LZ-120 (Labtech Engineering, Samut Prakan, Thailand).

### 2.3. Storage Conditions

The obtained darunavir-loaded granules were stored in two formats: as a bulk sample in a tightly closed container and as individually packed dosage units. For the preparation of dosage units, granules were portioned into 200.0 ± 0.1 mg units, corresponding to a nominal darunavir content of 100 mg per unit, and individually packed in heat-sealed foil-laminate sachets. Both bulk samples and individual dosage units were stored at 20 ± 2 °C. Relative humidity was not monitored during storage.

### 2.4. Preparation of a Reference PEG-Associated Darunavir Sample

To prepare a reference PEG-associated darunavir sample, amorphous darunavir was mixed with an excess of PEG 400 and stored at 20 ± 2 °C until a crystalline phase formed. The resulting sample was used as a reference material for differential scanning calorimetry and X-ray diffraction analysis.

### 2.5. Pharmacopoeial Evaluation of Darunavir-Loaded Granules

#### 2.5.1. Description

The appearance of the granules was assessed visually against a white background under diffuse lighting. Particle shape, surface characteristics, color uniformity and the presence of visible defects (e.g., cracks, unmelted regions, inclusions) were recorded.

#### 2.5.2. Particle Size Distribution (Sieve Analysis)

After pelletization, darunavir-loaded granules were sieved to isolate the target fraction of 2.0–2.5 mm, which was used for subsequent pharmacopoeial evaluation. Particle size distribution was determined by sieve analysis in accordance with standard pharmacopoeial methods. A digital electromagnetic sieve shaker BA200N and a set of analytical sieves (Cisa Cedaceria Industrial, Barcelona, Spain) with mesh sizes of 3.15, 2.80, 2.50, 2.00, 1.80, and 1.60 mm were employed.

#### 2.5.3. Determination of Disintegration

The disintegration of darunavir-loaded dosage units was determined in accordance with standard pharmacopoeial methods using an ERWEKA ZT 120 apparatus (ERWEKA GmbH, Hessen, Germany). The test was performed in phosphate buffer (pH 6.8) maintained at 37 ± 1 °C. A dosage unit was considered completely disintegrated when it lost its structural integrity in accordance with pharmacopoeial criteria. If a soft residue remained, it was considered acceptable only when it readily disintegrated upon gentle contact with a glass rod.

#### 2.5.4. Loss on Drying

Loss on drying was determined using a moisture analyzer AVG-60 (Gosmetr, Saint Petersburg, Russia). Samples of the target granule fraction were ground before analysis. A sample of 1.5 ± 0.01 g was dried at 105 ± 1 °C to constant weight. Loss on drying was calculated as the relative mass loss after drying.

#### 2.5.5. Uniformity of Dosage Units

Uniformity of dosage units was evaluated for individually packed darunavir-loaded granule units with a mass of 200.0 ± 0.1 mg, corresponding to a nominal darunavir content of 100 mg. Quantitative determination of darunavir was performed by UV spectrophotometry using a previously validated method [43]. The analysis was carried out in two independent laboratories using comparable analytical protocols.

Each dosage unit was transferred into a 200 mL volumetric flask, methanol was added, and the mixture was stirred until complete dissolution of the granules. The resulting solution was diluted to volume with methanol using Class A volumetric glassware and, if necessary, further diluted to a concentration within the method’s linear range. Darunavir content was calculated using a calibration curve constructed in methanol. Absorbance was measured at 268 nm using Cary 60 (Agilent Technologies, Santa Clara, CA, USA) and UNICO 2800 (United Products & Instruments, Inc., Dayton, NJ, USA) spectrophotometers. Dosage units were weighed using MS105/A (Mettler Toledo, Greifensee, Switzerland) and GH-120 (A&D Company, Tokyo, Japan) analytical balances.

### 2.6. Advanced Characterization of Darunavir-Loaded Granules

#### 2.6.1. Differential Scanning Calorimetry

Differential scanning calorimetry (DSC) was used to characterize the thermal behavior of amorphous darunavir, the initial polymeric premix, a reference PEG-associated darunavir sample, and darunavir-loaded granules. The initial polymeric premix and darunavir-loaded granules were analyzed immediately after preparation and after 24 months of storage. Measurements were performed using a Thermal Analysis System TGA/DSC 3+ (Mettler Toledo, Greifensee, Switzerland) over a temperature range of −120 to 200 °C at a heating rate of 10 °C/min under a nitrogen atmosphere with a flow rate of 50 mL/min. Each test was performed in triplicate, and results are reported as mean ± standard deviation.

Samples with a mass of 10 ± 3 mg were placed in aluminum crucibles (40 µL) and analyzed without prior drying. For each sample, two consecutive heating cycles were recorded. DSC analysis was performed for darunavir-loaded granules immediately after preparation and after two years of storage.

#### 2.6.2. X-Ray Diffraction Analysis

X-ray diffraction (XRD) analysis was used to characterize the solid-state structure of amorphous darunavir, the reference PEG-associated darunavir sample, and darunavir-loaded granules. Darunavir-loaded granules were analyzed immediately after preparation and after 24 months of storage. For each sample, measurements were performed in triplicate; representative diffractograms are presented in the figures.

X-ray diffraction was performed using a rotating copper anode source Rotaflex RU-200 (Rigaku, Tokyo, Japan) operating at 50 kV and 160 mA. The instrument was equipped with a horizontal wide-angle goniometer (Rigaku D/Max-RC), and diffraction patterns were recorded in Bragg–Brentano geometry. A scintillation counter was used to detect diffracted X-ray radiation.

The incident radiation was monochromatized using a secondary focusing monochromator mounted on the diffracted beam, consisting of a curved graphite single crystal. The wavelength of the monochromatized radiation was 1.542 Å. Diffraction patterns were recorded in both small-angle (1–10° 2θ) and wide-angle (3–60° 2θ) ranges. Measurements were performed in continuous scanning mode at a scanning rate of 4°/min with a step size of 0.04°.

#### 2.6.3. Dissolution Test

The dissolution test was performed using an ERWEKA DT 126 Light dissolution tester (ERWEKA GmbH, Langen, Germany) configured as USP Apparatus II. The dissolution medium consisted of 900 mL of 0.05 M phosphate buffer at pH 3.0 containing 2% Tween 20. The medium temperature was maintained at 37 ± 0.5 °C, and the paddle rotation speed was 75 rpm. The test conditions were selected based on previously reported methods for dosage forms containing darunavir [44].

For the granule samples, one dosage unit weighing 200 mg and corresponding to a nominal darunavir content of 100 mg was placed in each vessel. A 100 mg portion of the initial amorphous darunavir API was used as the control sample. Six replicates were tested for each experimental group.

Samples were withdrawn at 5, 10, 15, 30, 45, 60, and 90 min. At each time point, 5 mL of dissolution medium was withdrawn and replaced with an equal volume of fresh medium pre-equilibrated to the test temperature. Prior to quantitative analysis, the collected samples were filtered through 0.45 μm syringe filters.

Darunavir in the collected samples was quantified by UV spectrophotometry in accordance with a previously validated quantitative method for darunavir [43], adapted for the analysis of dissolution-test samples.

#### 2.6.4. HPLC Assessment of Darunavir

The HPLC assessment of darunavir was performed using a method based on a previously published stability-indicating HPLC method developed for the stability assessment of darunavir ethanolate tablets [45]. The chromatographic separation conditions were reproduced in accordance with the published method, while sample preparation was adapted to account for the composition of the investigated polymeric matrix.

The analysis was performed by high-performance liquid chromatography with UV detection using a "Stayer M" liquid chromatograph (SPE Akvilon LLC, Podolsk, Russia) equipped with a spectrophotometric detector. Chromatographic system control, data acquisition, and data processing were performed using MultiChrom software, version 3.5.

Chromatographic separation was performed under reversed-phase conditions on a Phenomenex Luna C18 column (250 × 4.6 mm, 5 μm) maintained at 35 °C. A mixture of water and acetonitrile (50:50, *v*/*v*) was used as the mobile phase under isocratic conditions. After each analytical cycle, the column was automatically washed with 90% acetonitrile. The flow rate was 1.0 mL/min, and the injection volume was 20 μL. UV detection was performed at 267 nm.

Granules produced at extrusion temperatures of 70 and 90 °C were examined immediately after preparation and after two years of storage. The initial darunavir API was used as a reference sample to determine the retention time of the main chromatographic peak. Each sample was analyzed in triplicate.

For sample preparation, the granules were first ground. A 30 mg portion, corresponding to approximately 15 mg of darunavir, was transferred to a 100 mL volumetric flask. Distilled water (7 mL) was added, and the mixture was shaken vigorously until a homogeneous suspension was obtained. Acetonitrile (20 mL) was then added, and the sample was sonicated for 10 min to desorb darunavir from the polymeric matrix. The volume was then adjusted to 100 mL with distilled water. A 1 mL aliquot of the resulting solution was transferred to a 10 mL volumetric flask and diluted to volume with the mobile phase. Before analysis, the solution was filtered through a 0.45 μm PTFE syringe filter.

The standard darunavir solution was prepared from the initial API. A 15 mg portion was transferred to a 100 mL volumetric flask, 50 mL of the mobile phase was added, and the solution was sonicated for 5 min until complete dissolution. The volume was then adjusted to 100 mL with the mobile phase. A 1 mL aliquot of the resulting solution was transferred to a 10 mL volumetric flask and diluted to volume with the mobile phase. Before analysis, the solution was filtered through a 0.45 μm PTFE filter.

The darunavir content in the investigated samples was calculated from the area of the main chromatographic peak relative to that of the standard darunavir solution, considering the sample weight and the nominal API content of the granules.

## 3. Results

### 3.1. Preparation of Darunavir-Loaded Granules by Hot-Melt Extrusion

Darunavir-loaded granules were prepared by HME using a previously developed polymeric premix designed for low-temperature extrusion processing. The extrusion temperature regimes were selected based on literature data regarding the thermal sensitivity of darunavir. According to the Merck Index [36], chemical degradation of amorphous darunavir begins at temperatures close to its glass transition temperature (70–80 °C), making it a critical model compound for assessing actual thermal exposure during HME.

Accordingly, extrusion was performed under two processing conditions corresponding to maximum recorded melt temperatures of 70 and 90 °C. A temperature of 70 °C was considered a conservative condition for thermal stability, enabling assessment of processability below the degradation threshold. In contrast, 90 °C exceeds the reported melting temperature of darunavir and falls within a range where the likelihood of thermally induced degradation increases. Thus, the selected temperature regimes enabled simultaneous assessment of the effect of short-term thermal exposure on API stability and analysis of changes in extrusion process parameters with increasing temperature.

The main extrusion parameters for both temperature conditions are summarized in Table 1. In all experiments, key process parameters [46], including melt pressure, torque, and flow behavior, reached steady-state values within the first few minutes of extrusion and remained stable thereafter. No signs of process instability or extruder overloading were observed.

At a melt temperature of 70 °C, extrusion was performed at a screw speed of 25 rpm and was associated with a high torque, reaching 41% of the maximum allowable value. Attempts to increase the screw speed under these conditions resulted in a sharp increase in torque, indicating limited melt mobility and operation near the upper boundary of the processing window. Despite this, a continuous and visually homogeneous extrudate was obtained, without signs of phase separation or incomplete homogenization.

At a melt temperature of 90 °C, the torque at the same screw speed (25 rpm) was only 2–3%, indicating significantly lower resistance to flow. Increasing the screw speed up to 100 rpm under these conditions led to an increase in torque to 23%, while stable extrusion was maintained. This behavior indicates a substantial expansion of the processing window and a pronounced temperature sensitivity of the rheological properties of the system [47].

After cooling to room temperature, the extruded filament exhibited brittleness and fractured under mechanical stress. Therefore, in all experiments, the extrudate was pelletized immediately after exiting the die using a strand pelletizer. The resulting granules did not exhibit sticking or deformation during cooling or subsequent storage at room temperature.

### 3.2. Pharmacopoeial Quality Attributes and Stability of Darunavir-Loaded Granules During Storage

#### 3.2.1. Stability Study Design

The study was conducted at four time points: immediately after preparation (0 months) and after 6, 12, and 24 months of storage. At each time point, basic pharmacopoeial quality attributes were evaluated, including appearance, particle size distribution, disintegration, loss on drying, and uniformity of dosage units.

#### 3.2.2. Description

**Figure 1:**
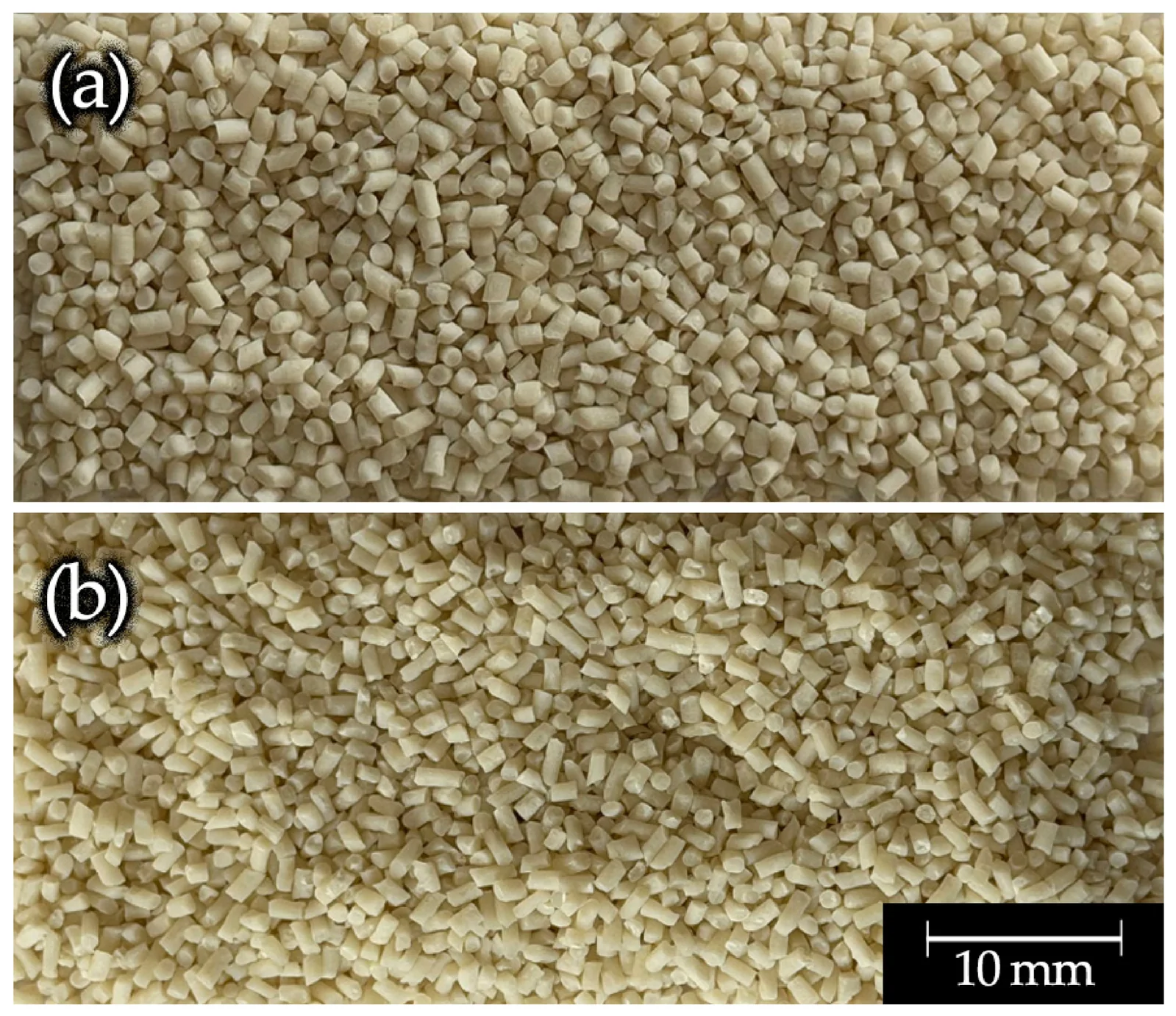
Darunavir-loaded granules prepared at 70 °C (**a**) and 90 °C (**b**).

The obtained darunavir-loaded granules were solid, creamy white particles with uniform coloration and a smooth surface (Figure 1). The material was visually homogeneous, without visible inclusions, cracks, or signs of phase separation. The granules remained free-flowing and retained their regular shape during storage.

The samples were odorless. No changes in organoleptic properties were observed at any storage time point (0, 6, 12, and 24 months). No signs of agglomeration, exudation of low-molecular-weight components, or deterioration of flowability were detected, either during bulk storage or in individual single-dose packaging.

#### 3.2.3. Particle Size Distribution

After pelletization, the resulting granules were sieved to obtain a target fraction with a particle size range of 2.0–2.5 mm, which was used for subsequent pharmacopoeial evaluation.

At all storage time points (0, 6, 12, and 24 months), the particle size characteristics of the granules remained unchanged. No formation of fine particles, oversized fractions, or signs of agglomeration were observed. The granules retained consistent size and shape throughout the entire storage period.

#### 3.2.4. Disintegration

At all storage time points (0, 6, 12, and 24 months), the granules completely lost their structural integrity within the limits specified by pharmacopoeial requirements. In all cases, the test resulted in a soft residue that readily disintegrated upon gentle contact with a glass rod [48].

The appearance and behavior of this residue remained consistent throughout the entire storage period. No formation of a hard, non-disintegrating residue was observed.

#### 3.2.5. Loss on Drying

Loss on drying of darunavir-loaded granules remained unchanged across all storage time points. However, a consistent difference between samples prepared at different extrusion temperatures was observed: granules obtained at 70 °C exhibited a loss on drying of 0.33 ± 0.09%, whereas those prepared at 90 °C showed a lower value of 0.27 ± 0.08% [49].

Given the identical storage conditions and the same composition of the initial materials, this difference may be attributed to the extrusion temperature and likely reflects variations in the amount of sorbed moisture in the granules.

#### 3.2.6. Uniformity of Dosage Units

The uniformity of dosage units of darunavir-loaded granules was evaluated in accordance with pharmacopoeial requirements using the acceptance value (AV) for individual dosage units with a nominal darunavir content of 100 mg (Stage 1, *n* = 10) [50]. The AV was calculated for granules prepared at extrusion temperatures of 70 and 90 °C at all storage time points (0, 6, 12, and 24 months).

In all investigated series, the calculated AVs were well below the pharmacopoeial acceptance limit (AV ≤ 15.0), confirming compliance with the requirements for uniformity of dosage units (Table 2). The AVs ranged from 6.07 to 7.86 and showed no systematic changes during storage. No significant differences were observed between granules prepared at different extrusion temperatures.

These results demonstrate that darunavir-loaded granules retained acceptable basic pharmacopoeial quality attributes throughout 24 months of storage. Appearance, particle size characteristics, disintegration behavior, loss on drying, and dosage-unit uniformity remained stable. Thus, routine quality-control procedures comparable to those available in pharmacy compounding practice did not indicate deterioration of the formulation.

### 3.3. Solid-State Changes and Crystallinity of Darunavir in Granules

#### 3.3.1. Differential Scanning Calorimetry

**Figure 2:**
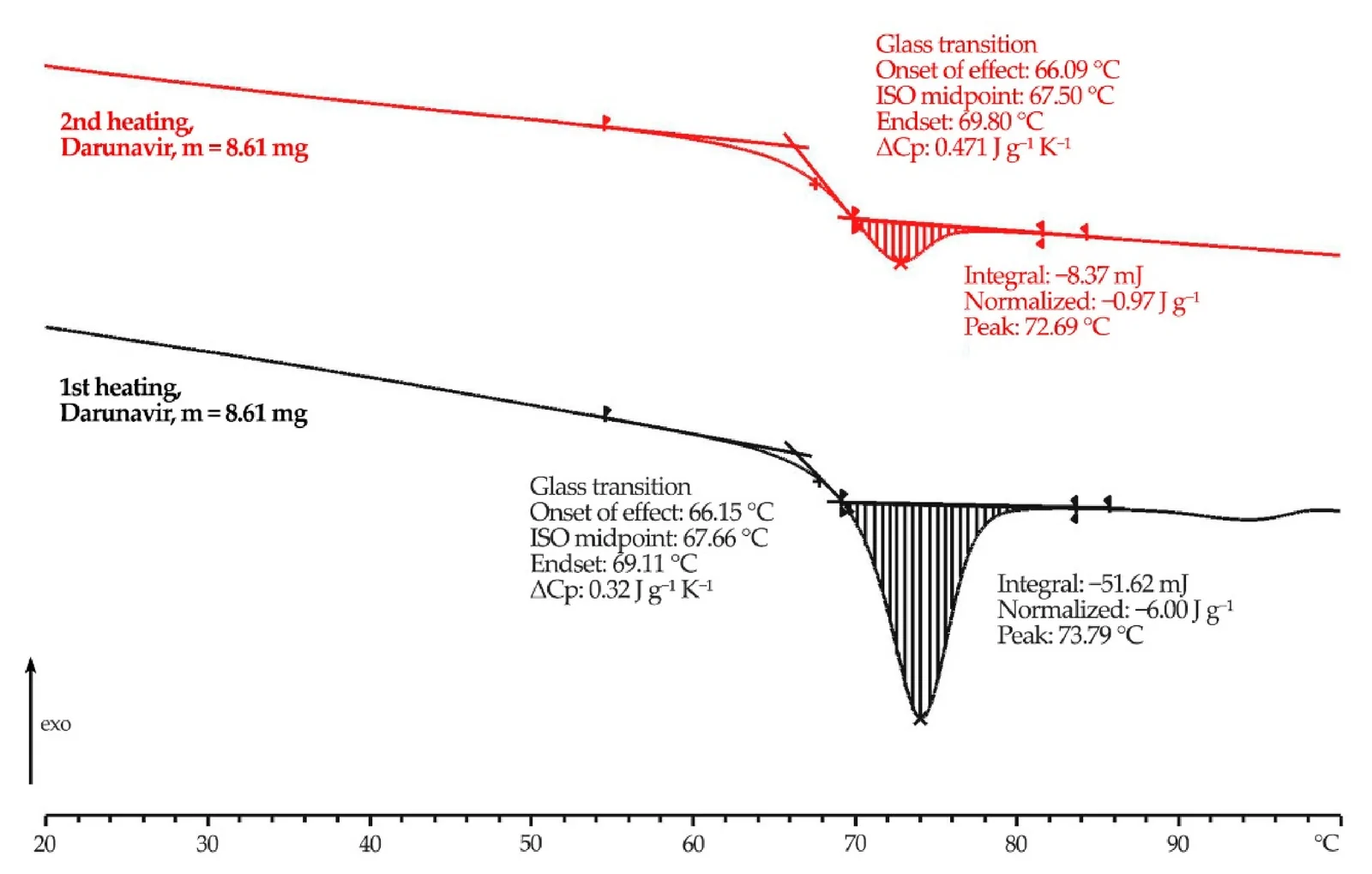
DSC thermograms of the initial darunavir.

DSC analysis of the initial darunavir sample revealed a glass transition at approximately 67.6 ± 0.2 °C and an endothermic melting event at 73.8 ± 0.1 °C during the first heating cycle (Figure 2). The relatively low melting enthalpy (6.0 ± 0.4 J/g), together with the disappearance of the melting event during the second heating cycle, indicates the presence of only a minor residual crystalline fraction in the starting material [26,51].

**Figure 3:**
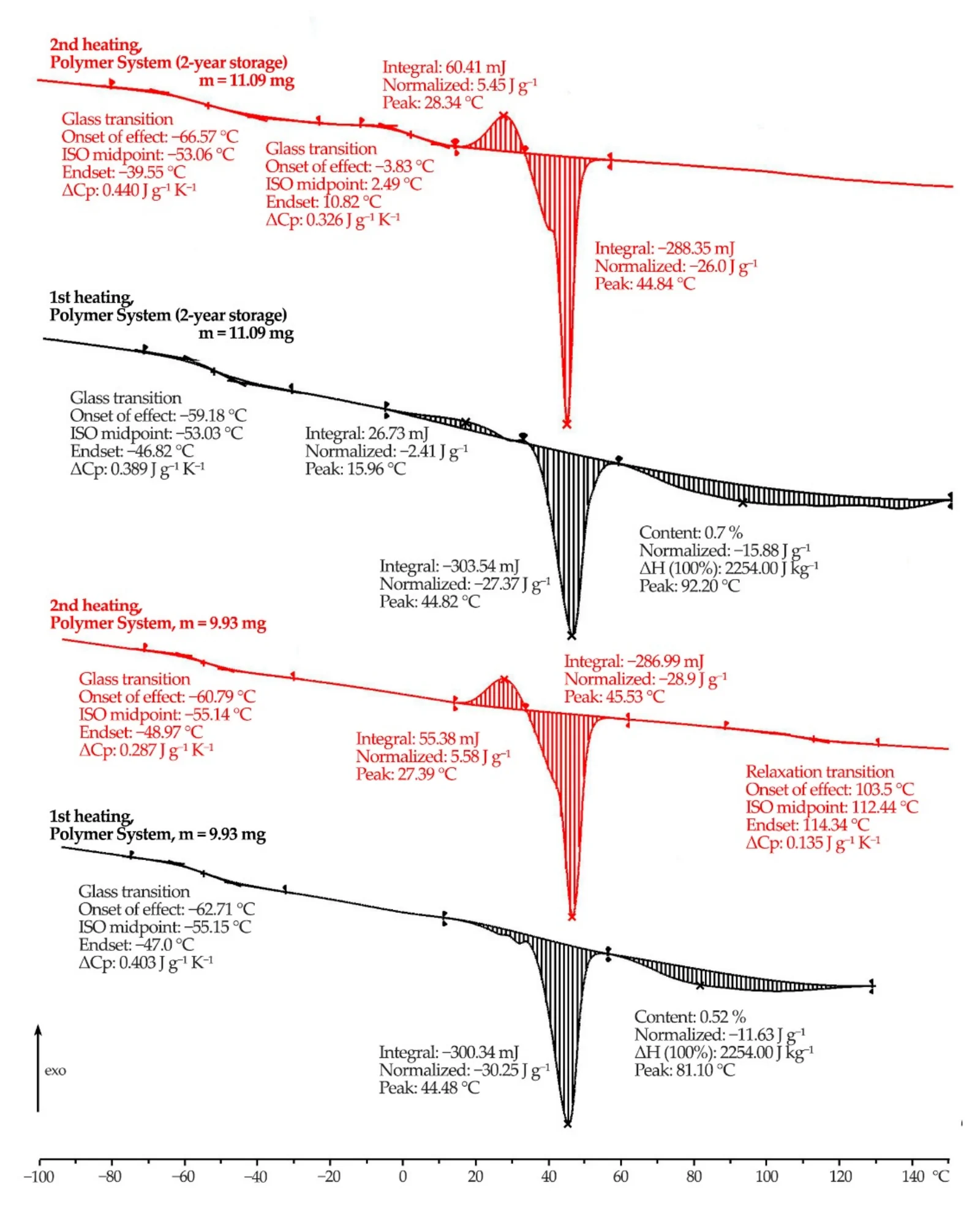
DSC thermograms of the initial polymeric system recorded immediately after preparation [14] and after two years of storage.

During the first heating cycle, the DSC thermograms of the initial polymeric system and stored samples showed the same principal thermal events (Figure 3). A low-temperature glass transition was observed at −55 ± 0.2 °C and −53 ± 0.2 °C, respectively. This was followed by an endothermic peak at 44.6 ± 0.12 °C, which corresponded to the melting of crystalline PEG 1500 [52]. Above 80 °C, a broad endothermic event was observed. It was attributed to the desorption of bound moisture (<1%) due to the hygroscopic nature of PVP K-29/32 and PEG.

The second-heating thermograms also showed comparable thermal behavior. Cold crystallization was followed by the melting of PEG 1500. In the stored samples, an additional weak glass transition was detected at around 2.5 °C. It was attributed to a fraction of free PEG 1500 that remained amorphous during cooling. Overall, the close agreement between the principal thermal events indicates that no substantial changes in the phase state or thermal behavior of the polymeric system occurred during two years of storage.

**Figure 4:**
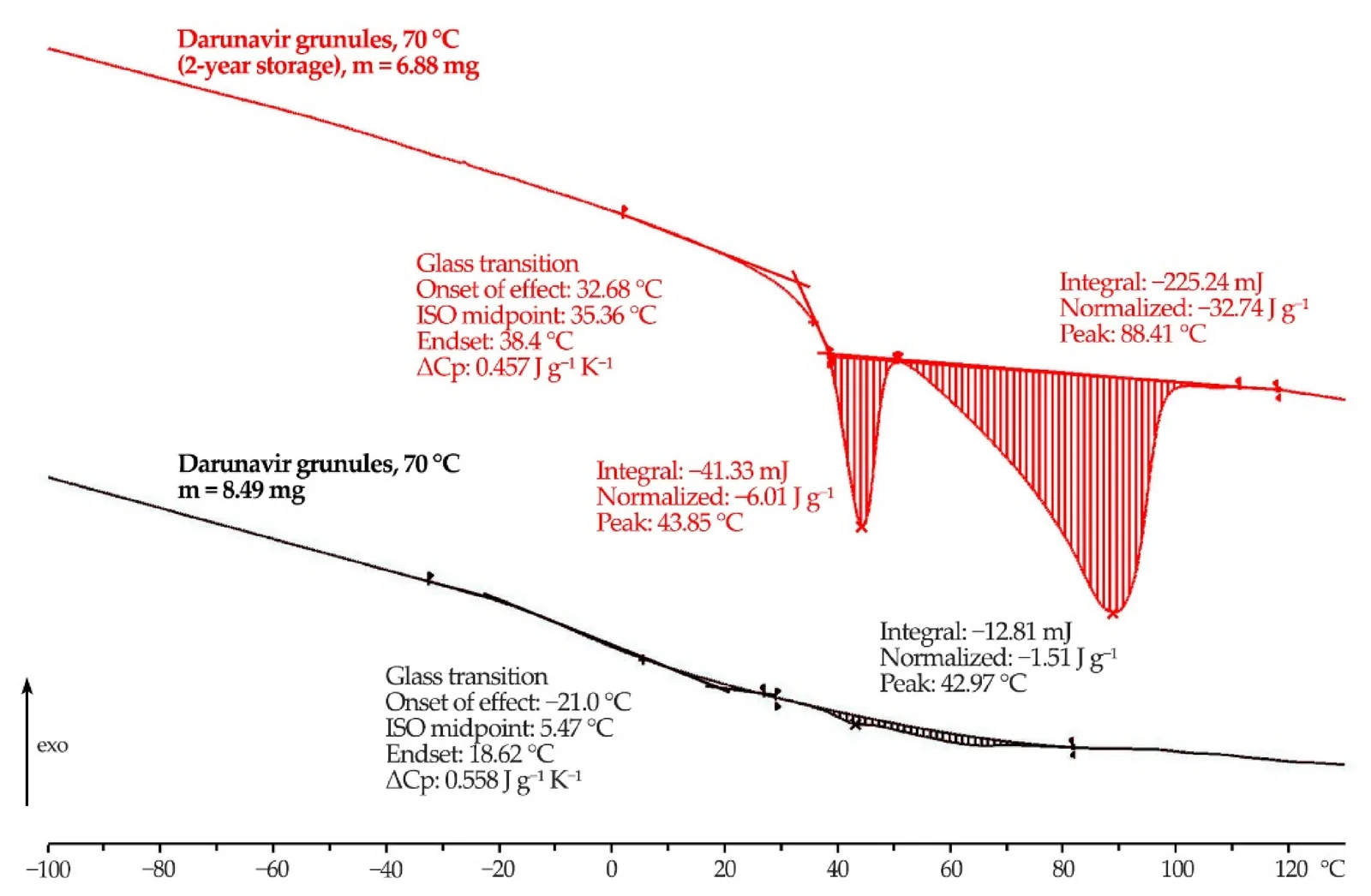
DSC thermograms of darunavir-loaded granules prepared at 70 °C: immediately after preparation (black) and after two years of storage (red).

For darunavir-loaded granules obtained at an extrusion temperature of 70 °C, the DSC curve recorded immediately after preparation did not show a distinct glass transition in the range of 70–80 °C corresponding to amorphous darunavir, indicating the quasi-homogeneous nature of the system (Figure 4) [23,51,53]. The thermograms were characterized by a broad glass transition with a midpoint temperature of approximately 5.5 ± 0.2 °C and a minor endothermic peak at 43 ± 0.3 °C, attributed to the melting of PEG 1500. After two years of storage, several changes were observed. A more distinct glass transition was observed in the range of 33–38 °C, the melting peak of PEG 1500 at 44 °C became more pronounced, and a new endothermic event emerged with a peak at 88 ± 0.5 °C.

**Figure 5:**
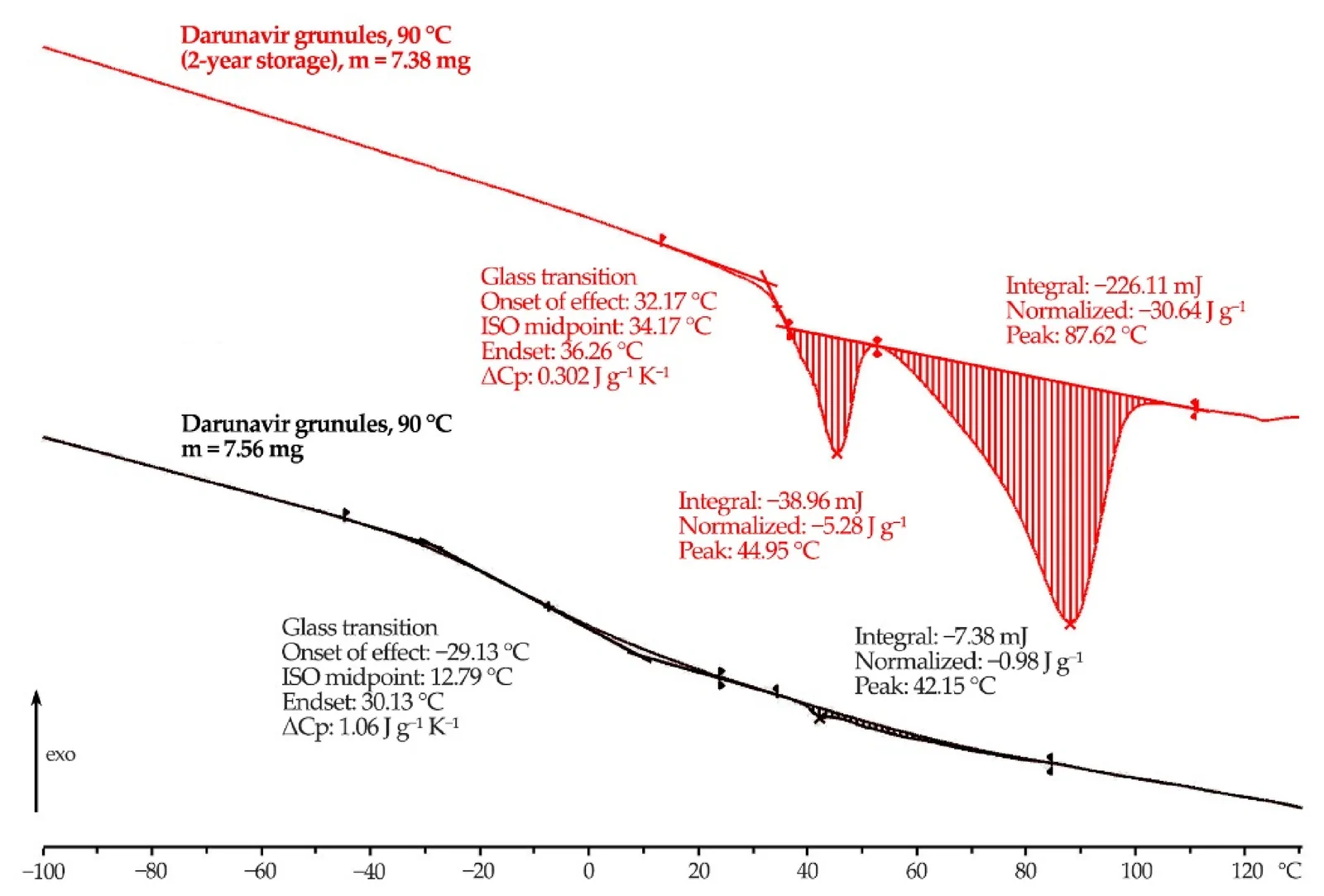
DSC thermograms of darunavir-loaded granules prepared at 90 °C: immediately after preparation (black) and after two years of storage (red).

A similar behavior was observed for darunavir-loaded granules prepared at an extrusion temperature of 90 °C. Immediately after preparation, the DSC curve exhibited a broad glass transition with a midpoint temperature of approximately 12.8 ± 0.3 °C and a weak endothermic peak at 42 ± 0.2 °C corresponding to the melting of the crystalline phase of PEG 1500 (Figure 5). After two years of storage, a more distinct glass transition in the range of 32–36 °C was observed, the melting peak of PEG 1500 at 44 °C became more pronounced, and a new endothermic event appeared with a peak at 88 ± 0.4 °C. The characteristic temperatures of the thermal events for samples obtained at 70 and 90 °C were qualitatively similar. Thus, samples obtained at 70 and 90 °C showed qualitatively similar changes in thermal behavior after two years of storage.

**Figure 6:**
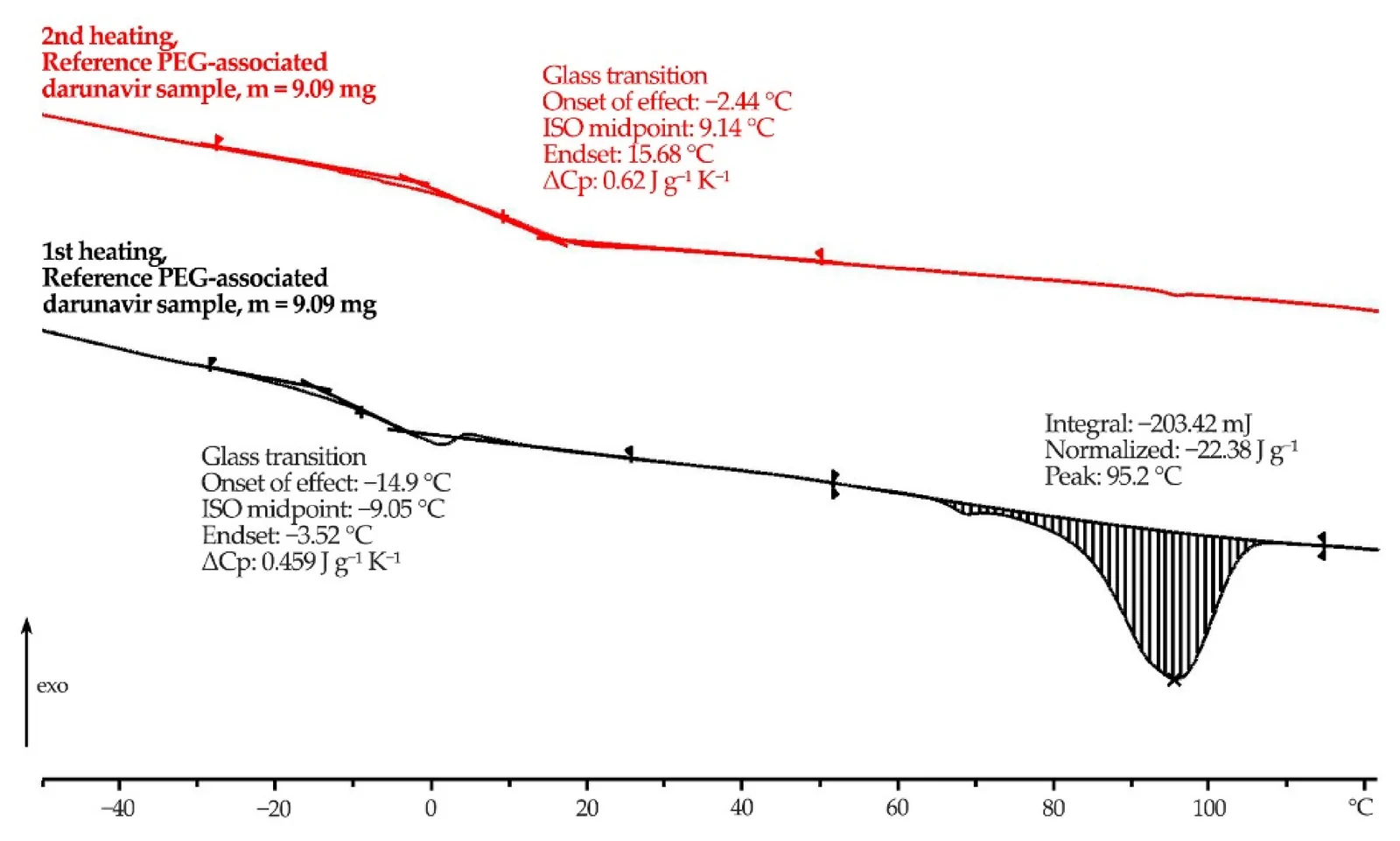
DSC thermograms of the reference PEG-associated darunavir sample.

To clarify the nature of the thermal events observed in the stored granules, a reference PEG-associated darunavir sample was prepared and analyzed by DSC (Figure 6).

In contrast to the initial darunavir, the reference PEG-associated darunavir sample exhibited a pronounced endothermic event with a peak temperature of 95.2 ± 1.1 °C and a melting enthalpy of 22.4 ± 1.8 J/g. The disappearance of this event during the second heating cycle indicates melting of the crystalline phase during the first heating cycle. Notably, the temperature of this transition is substantially higher than the melting event observed for the initial darunavir (~74 °C) and closer to the thermal event detected in stored granules (~88 °C), supporting the presence of a PEG-associated crystalline phase [34,35,40].

Thus, the DSC results indicate a similar pattern of thermal behavior in darunavir-loaded granules prepared at different extrusion temperatures. Comparison with the reference PEG-associated darunavir sample suggests that the newly observed thermal event may be associated with the formation of a PEG-associated crystalline phase during storage. Further support for this phase assignment was therefore sought by X-ray diffraction analysis.

#### 3.3.2. X-Ray Diffraction Analysis

**Figure 7:**
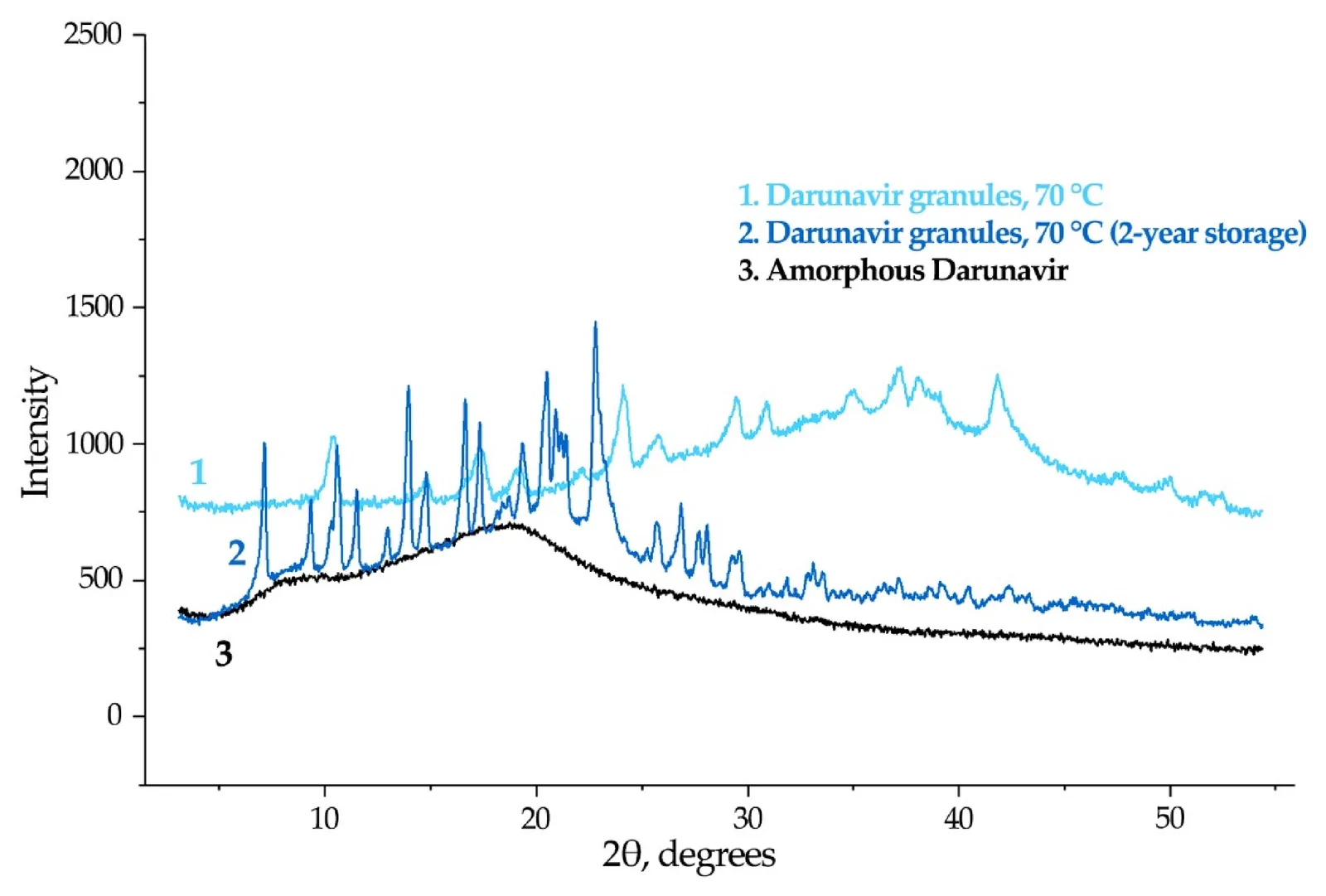
X-ray diffraction patterns of amorphous darunavir and darunavir-loaded granules prepared at 70 °C: immediately after preparation and after two years of storage.

X-ray diffraction (XRD) analysis was performed for darunavir-loaded granules prepared at a melt temperature of 70 °C. Diffraction patterns were recorded immediately after preparation and after two years of storage (Figure 7). For proper interpretation, the diffraction pattern of the initial darunavir was used as a reference; it exhibited an amorphous profile with two weak maxima at 8° and 19°, confirming its amorphous state. The diffraction pattern of the composition recorded immediately after preparation was predominantly amorphous but showed a set of weak reflections at angles corresponding both to crystalline PEG 1500 and to additional, previously absent reflections. The presence of these peaks indicates the formation of an additional crystalline phase within the system [26,50]. After two years of storage, the number of reflections increased significantly, with enhanced intensity observed in the angular regions corresponding to the maximum of the amorphous darunavir profile. This indicates substantial growth of an additional crystalline phase during storage.

**Figure 8:**
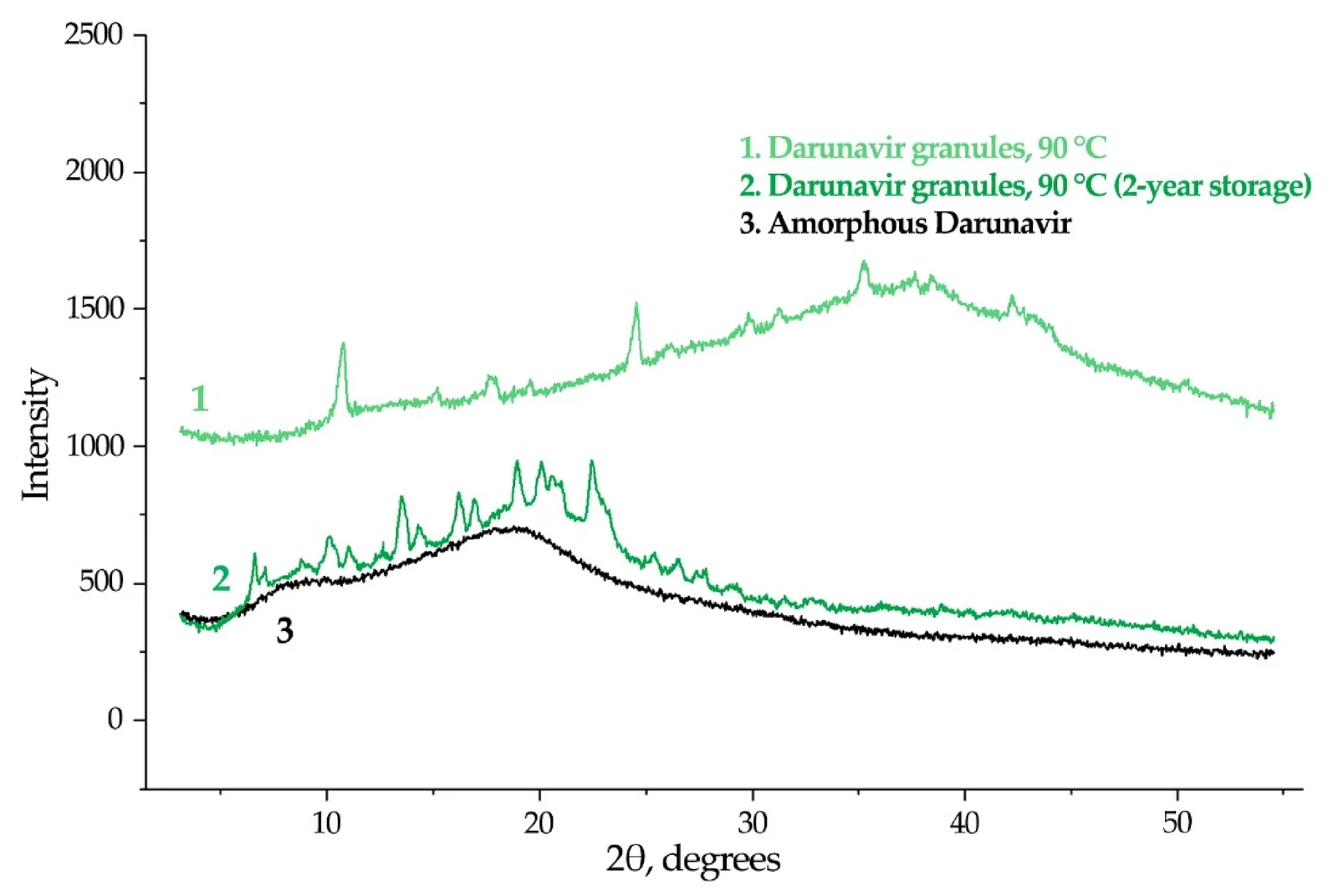
X-ray diffraction patterns of amorphous darunavir and darunavir-loaded granules prepared at 90 °C: immediately after preparation and after two years of storage.

A similar analysis was performed for granules prepared at a melt temperature of 90 °C (Figure 8). The diffraction pattern recorded immediately after preparation was also characterized by a predominantly amorphous halo with a limited number of weak diffraction reflections. After storage, an increase in the number of reflections was observed in the angular regions corresponding to darunavir, indicating the formation of a new crystalline phase during storage.

Comparison of the diffraction patterns of granules prepared at 70 and 90 °C revealed qualitatively similar solid-state changes after storage. However, the number and intensity of reflections were lower for the granules prepared at 90 °C, indicating differences in the extent and/or structural organization of the crystalline phase between the two systems.

The emergence of new endothermic peaks in the DSC thermograms and additional reflections in the XRD patterns after storage of darunavir-loaded granules indicate the formation of a new crystalline phase [23,26,51]. It is therefore reasonable to assume that, upon mixing amorphous darunavir with the polymeric system—where PEG 400 serves as the main binding component—darunavir dissolves in the polyethylene glycol phase, leading to the formation of crystalline solvates [40]. According to the literature [34], endothermic peaks corresponding to different crystalline solvates of amorphous darunavir are observed in the temperature range of 65–118 °C, which is consistent with the present findings.

**Figure 9:**
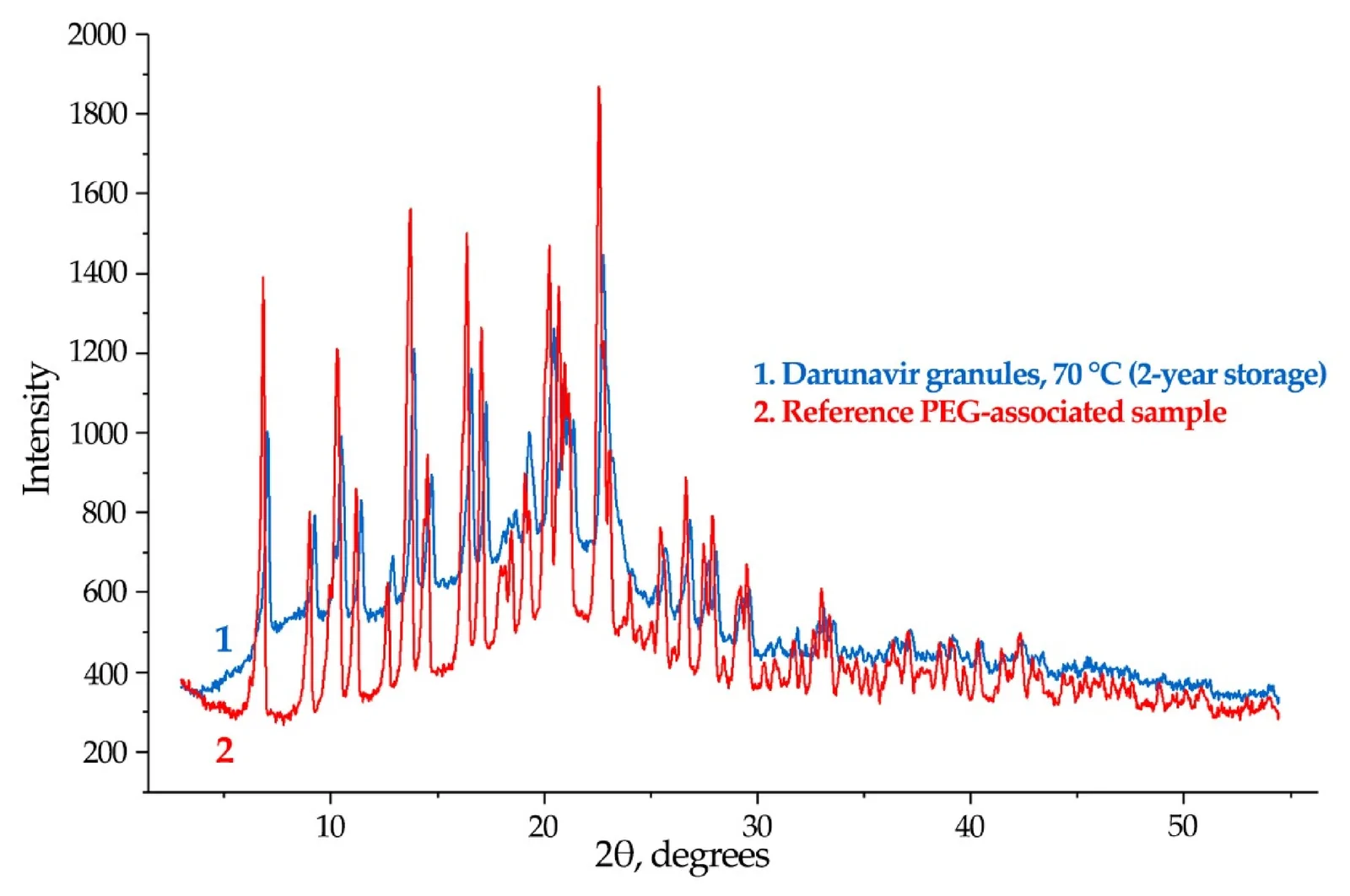
Comparison of X-ray diffraction patterns of darunavir-loaded granules prepared at an extrusion temperature of 70 °C after two years of storage and the reference PEG-associated darunavir sample.

**Figure 10:**
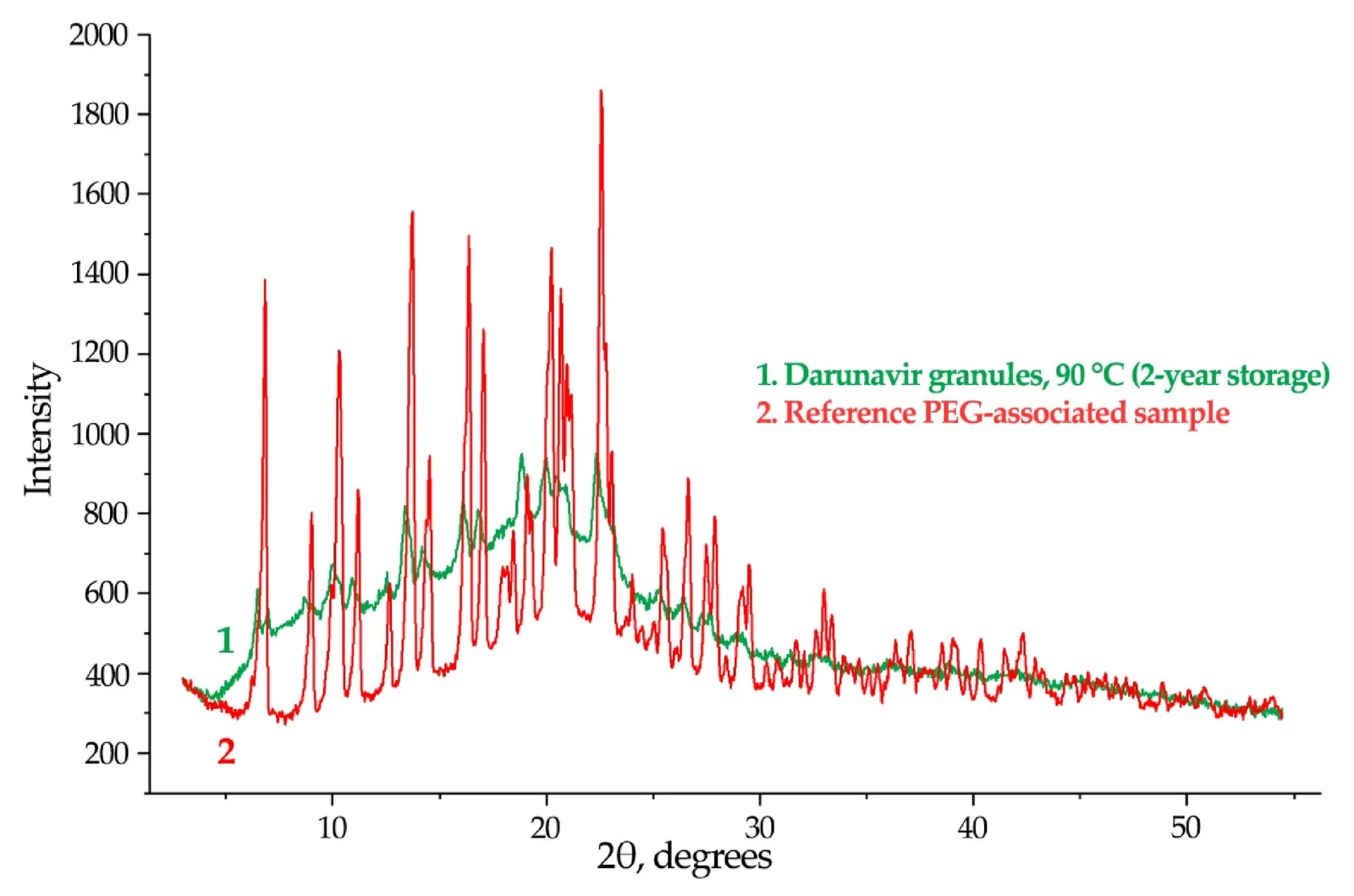
Comparison of X-ray diffraction patterns of darunavir-loaded granules prepared at an extrusion temperature of 90 °C after two years of storage and the reference PEG-associated darunavir sample.

As in the DSC analysis, a reference PEG-associated darunavir sample was characterized by X-ray diffraction to further clarify the nature of the crystalline phase formed during storage. Comparison of the diffraction patterns of darunavir-loaded granules obtained at extrusion temperatures of 70 and 90 °C after two years of storage with that of the reference PEG-associated darunavir sample revealed a close correspondence of the characteristic reflection positions (Figure 9 and Figure 10). The relative peak intensities showed better agreement with the reference sample for the granules prepared at 70 °C, whereas the granules prepared at 90 °C exhibited the same set of characteristic reflections but with more pronounced differences in relative intensities. These results indicate that a similar PEG-associated crystalline phase was present in both systems, while the extent and/or microstructural organization of this phase may differ with extrusion temperature [17].

### 3.4. Dissolution Test

**Figure 11:**
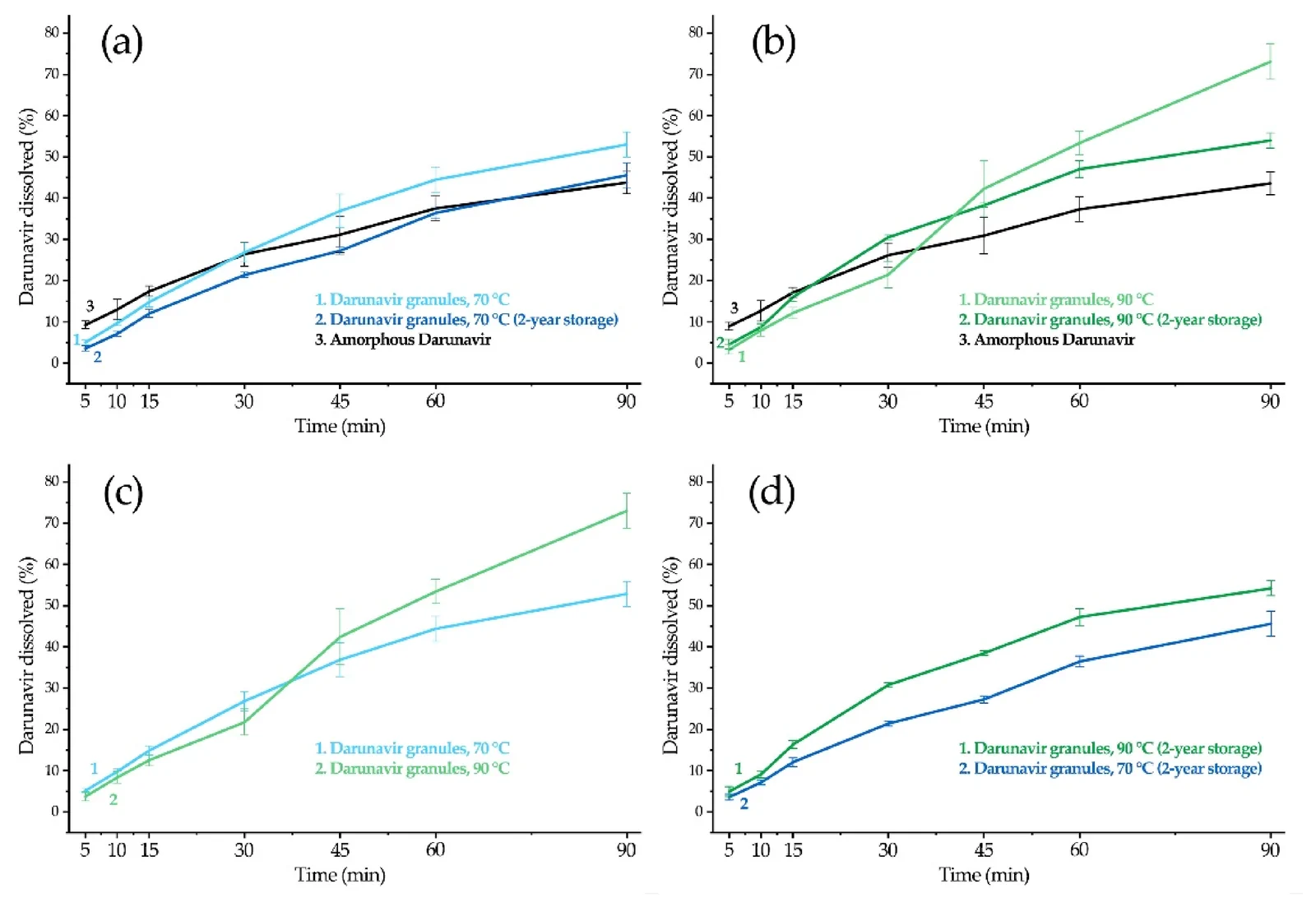
Dissolution profiles of darunavir from the initial amorphous API and HME granules: initial amorphous darunavir API and granules produced at 70 °C, tested immediately after preparation and after two years of storage (**a**); initial amorphous darunavir API and granules produced at 90 °C, tested immediately after preparation and after two years of storage (**b**); comparison of granules produced at 70 and 90 °C immediately after preparation (**c**); comparison of granules produced at 70 and 90 °C after two years of storage (**d**). Data are presented as mean ± SD, *n* = 6.

To assess the possible influence of the identified solid-state changes in darunavir on dosage-form properties, dissolution testing was performed on the initial amorphous darunavir API and granules produced at 70 and 90 °C, both immediately after preparation and after two years of storage (Figure 11).

None of the investigated samples reached a dissolution plateau within 90 min. For the initial amorphous darunavir API, the amount dissolved after 30, 60, and 90 min was 26.47 ± 2.94, 37.55 ± 3.03, and 43.83 ± 2.73%, respectively. During the test, darunavir particles agglomerated and formed a hydrated outer layer, while dry material remained inside the aggregates. This reduced contact with the dissolution medium.

For granules extruded at 70 °C and tested immediately after preparation, the amount of darunavir dissolved after 30, 60, and 90 min was 26.89 ± 2.33, 44.51 ± 3.06, and 53.07 ± 3.03%, respectively. After two years of storage, the corresponding values were 21.43 ± 0.64, 36.47 ± 1.28, and 45.61 ± 3.03%.

For granules produced at 90 °C, the amount of darunavir dissolved after 30, 60, and 90 min was 21.78 ± 3.17, 53.55 ± 2.92, and 73.19 ± 4.24%, respectively, for samples tested immediately after preparation, compared with 30.81 ± 0.55, 47.23 ± 2.08, and 54.19 ± 1.8% after storage.

Thus, differences in dissolution profiles were observed between samples tested immediately after preparation and after two years of storage, with a more pronounced overall difference for granules produced at 90 °C. At 90 min, the stored granules from both groups showed darunavir dissolution comparable to or greater than that of the initial amorphous API.

### 3.5. HPLC Assessment of Darunavir

HPLC analysis showed that the retention time of the main darunavir peak was essentially unchanged across all investigated samples. The retention time was 8.05 min for the reference darunavir API and ranged from 8.05 to 8.07 min for granules produced at 70 and 90 °C, both immediately after preparation and after two years of storage.

The determined darunavir content was 97.40 ± 0.69% and 98.58 ± 0.7% of the nominal value for granules produced at 70 °C and analyzed immediately after preparation and after two years of storage, respectively. For granules produced at 90 °C, the corresponding values were 98.82 ± 0.7% and 100.47 ± 0.71%, respectively.

These data were consistent with the quantitative results obtained by UV spectrophotometry and confirmed preservation of darunavir content after extrusion and long-term storage.

## 4. Scope and Limitations of the Study

The original study design did not include dissolution testing or chromatographic analysis. To perform these additional investigations, a new batch of darunavir-loaded granules was prepared under conditions identical to those used in the original study, including the same materials and laboratory setting. Accordingly, the samples evaluated immediately after preparation in the dissolution and HPLC studies were produced as a separate batch and were not the same batch samples as those examined after long-term storage.

Quantitative determination of darunavir by UV spectrophotometry was used to assess dosage-unit uniformity after complete dissolution of the samples in methanol. This method was not considered evidence of the chemical stability of darunavir and was not used to assess potential degradation products.

The formation of a new crystalline phase was based on differential scanning calorimetry and X-ray diffraction data, using a separately prepared PEG-associated darunavir sample as reference material. Despite the high degree of correspondence between the obtained data, the present study did not include a complete crystallographic characterization of the new phase, including the determination of its stoichiometry and detailed crystal structure [27]. Therefore, throughout the following text, this phase is considered a PEG-associated crystalline phase of darunavir based on DSC and XRD data, rather than a fully structurally characterized solvated form of darunavir [33,34].

Notably, a crystalline propylene glycol solvate of darunavir has previously been described [35]. Its reported DSC profile showed a single endothermic peak at 94.93 °C, which is very close to the endothermic event observed for the reference PEG-associated sample in the present study (95.2 ± 1.1 °C). In addition, the reported X-ray diffraction pattern of the propylene glycol solvate showed substantial similarity to the X-ray diffraction pattern of the reference PEG-associated sample.

This study was conducted using a single model API and a laboratory-scale twin-screw extruder. Therefore, the obtained results should not be automatically extrapolated to other APIs, alternative polymer-matrix compositions, or the feasibility of implementing HME in pharmacy-compounding settings. These aspects require further technological development and regulatory assessment.

## 5. Discussion

The present study demonstrated that darunavir-loaded granules prepared by low-temperature hot-melt extrusion using a previously developed polymeric premix maintained acceptable pharmacopoeial quality attributes throughout the entire storage period. Samples produced at both 70 and 90 °C retained comparable characteristics throughout storage. Appearance, particle-size distribution, loss on drying, disintegration, and dosage-unit uniformity remained within the established acceptance criteria and showed no significant changes over time. HPLC analysis confirmed comparable darunavir content in all investigated samples. No evidence of substantial chemical degradation was observed after extrusion at either processing temperature or after long-term storage.

However, differential scanning calorimetry and X-ray diffraction revealed API solid-state changes and the formation of a new crystalline phase [54].

These findings indicate that the preservation of routine quality attributes does not necessarily imply the preservation of the physicochemical state of the active pharmaceutical ingredient. This observation is particularly relevant for amorphous systems, in which structural relaxation, recrystallization, and other solid-state transformation processes may occur without producing detectable changes in parameters traditionally used for pharmacopoeial quality control [55].

Differences in dissolution profiles were also observed between samples tested immediately after preparation and after long-term storage. Granules tested immediately after preparation generally showed greater darunavir release than the corresponding stored samples, with the difference being more pronounced for granules extruded at 90 °C. Thus, the observed solid-state changes were associated with altered darunavir release from the granules [56].

Accordingly, pharmacopoeial stability of a dosage form and solid-state stability of the API should be regarded as related but distinct characteristics.

Comparison of the DSC and XRD results with those obtained for the reference PEG-associated sample indicates the formation of a PEG-associated crystalline phase of darunavir during storage. This interpretation is consistent with previous laser microinterferometry studies demonstrating that darunavir is capable of dissolving in polyol-containing media, including PEG 400, followed by the formation of crystalline solvated structures. Since PEG 400 is one of the key components of the investigated polymeric premix, the observed recrystallization may represent a thermodynamically favorable pathway of structural reorganization of the API within the system. Importantly, evidence of new crystalline-phase formation was observed in granules produced at both 70 and 90 °C. This finding suggests that the long-term behavior of the system depends more on the composition and internal organization of the polymer matrix than on the extrusion temperature [57,58,59].

These results are relevant not only to hot-melt extrusion technology itself but also to the broader challenge of adapting industrial pharmaceutical technologies for pharmacy compounding [60,61,62,63]. Accordingly, the development of compounded medicines and the justification of their stability should rely on the same physicochemical principles that apply to industrially manufactured drug products. Conversely, technological platforms based on API amorphization, polymer matrices, hot-melt extrusion, or additive manufacturing require equally rigorous assessment of component compatibility and potential pathways of solid-state transformation, and their possible impact on drug release [57,58].

The relatively short shelf life typically assigned to compounded preparations should not be considered sufficient justification for omitting such investigations. As demonstrated in the present study, API solid-state changes may occur without corresponding changes in routine pharmacopoeial quality attributes and may affect drug release. Therefore, the relevance of such transformations should be considered during formulation development and stability assessment even when relatively short storage periods are anticipated.

For dosage forms based on solid dispersions and other systems in which the solid state of the API may be unstable, routine pharmacopoeial quality control used in pharmacy compounding may be insufficient to justify stability and establish shelf life. In such cases, the stability program should include additional physicochemical and analytical studies selected on a risk-based basis. Depending on the characteristics of the API and formulation, these may include solid-state characterization, dissolution testing, chromatographic analysis, or other appropriate methods.

Conducting such studies in every individual pharmacy may not be feasible because of the cost of analytical instrumentation, the complexity of the methods, and the need for appropriately trained personnel. For complex compounded dosage forms, the development of an analytical strategy and stability assessment may therefore be outsourced to an appropriately qualified and, where required by national legislation, authorized laboratory, university-based center, or other organization possessing the necessary infrastructure and expertise.

Under such an approach, comprehensive characterization would be performed primarily during formulation development and shelf-life justification, whereas routine quality control at the pharmacy could rely on a defined set of pharmacopoeial and technological quality attributes established through the preceding stability study. The specific requirements for such an approach would necessarily depend on the national regulatory framework governing pharmacy compounding.

Overall, the results of the present study indicate that the development of dosage forms intended for pharmacy compounding should consider not only the technological feasibility of manufacturing and compliance with routine quality-control procedures used in compounding practice but also the long-term behavior of the API within the formulation.

## 6. Conclusions

The present study investigates solid-state changes in amorphous darunavir incorporated into a polymeric matrix designed for low-temperature hot-melt extrusion. The results show that the previously developed polymeric premix enables the preparation of darunavir-loaded granules by HME at 70 and 90 °C while maintaining process robustness and acceptable pharmacopoeial quality attributes. Differences in extrusion temperature do not substantially affect the pharmacopoeial quality attributes of the granules throughout the storage period.

Despite the preservation of appearance, particle-size distribution, disintegration, loss on drying, and dosage-unit uniformity, DSC and X-ray diffraction reveal API solid-state changes during storage. In both investigated systems, the formation of a new PEG-associated crystalline phase of darunavir is observed. These solid-state changes were accompanied by changes in the dissolution profiles of the granules, which were more pronounced for samples prepared at 90 °C.

These results demonstrate that compliance with routine quality-control procedures used in pharmacy compounding practice does not exclude solid-state transformation of the API. Accordingly, pharmacopoeial stability of a dosage form and solid-state stability of the active pharmaceutical ingredient are not equivalent characteristics and should be considered separately during the development of drug products containing amorphous APIs.

The findings of this study are relevant to the adaptation of hot-melt extrusion and other advanced manufacturing platforms to pharmacy compounding. The implementation of such technologies in pharmacy compounding practice requires not only technological reproducibility of the process but also a scientifically justified assessment of component compatibility, dosage-form stability, and potential pathways of API solid-state transformation.

Overall, this work shows that conventional routine quality attributes are insufficient as the sole basis for stability justification in HME-derived systems intended for pharmacy compounding. Solid-state characterization should therefore be considered an important component of risk assessment for compounded formulations containing amorphous or solvatomorphic APIs, particularly when routine quality control is used to justify stability.

## Figures and Tables

**Table 1:** Extrusion parameters for the preparation of darunavir-loaded granules at 70 and 90 °C.

Maximum Barrel Temperature (°C)	Die Temperature (°C)	Maximum Recorded Melt Temperature (Measured), °C	Screw Speed (rpm)	Torque (% of Maximum)	Melt Pressure (bar)	Observations
70	70	70	25	41	<10	Homogeneous extrudate
90	90	89	25	3	<10	Homogeneous extrudate

**Table 2:** Uniformity of dosage units of darunavir-loaded granules expressed as Acceptance Value (AV). X—mean darunavir content (% of nominal); s—standard deviation; M—reference value; k—acceptability constant (k = 2.4 for *n* = 10); AV—acceptance value calculated as AV = |M − X| + k·s. Acceptance criterion: AV ≤ 15.0.

Sample	Time Point	X (%)	s	M (%)	k	AV (%)	Criterion
70	0 mo	102.23	2.70	101.5	2.4	7.21	AV ≤ 15.0
70	6 mo	98.76	3.25	98.76	2.4	7.80	AV ≤ 15.0
70	12 mo	100.23	2.65	100.23	2.4	6.36	AV ≤ 15.0
70	24 mo	102.43	2.88	101.5	2.4	7.84	AV ≤ 15.0
90	0 mo	99.93	2.53	99.93	2.4	6.07	AV ≤ 15.0
90	6 mo	103.29	2.53	101.5	2.4	7.86	AV ≤ 15.0
90	12 mo	98.11	2.58	98.5	2.4	6.58	AV ≤ 15.0
90	24 mo	101.53	2.89	101.5	2.4	6.97	AV ≤ 15.0

## Data Availability

The original contributions presented in this study are included in the article. Further inquiries can be directed to the corresponding author.

## Abbreviations

The following abbreviations are used in this manuscript:

API	Active Pharmaceutical Ingredient
AV	Acceptance Value
DSC	Differential Scanning Calorimetry
EP	European Pharmacopoeia
FDM	Fused Deposition Modeling
HME	Hot-Melt Extrusion
HPC	Hydroxypropylcellulose
HPLC	High-Performance Liquid Chromatography
PEG	Polyethylene Glycol
PVP	Polyvinylpyrrolidone
USP	United States Pharmacopeia
UV	Ultraviolet
XRD	X-ray Diffraction

## References

[1] Patil H., Vemula S.K., Narala S., Lakkala P., Munnangi S.R., Narala N., Jara M.O., Williams R.O., Terefe H., Repka M.A. (2024). Hot-Melt Extrusion: From Theory to Application in Pharmaceutical Formulation—Where Are We Now?. AAPS PharmSciTech.

[2] Repka M.A., Bandari S., Kallakunta V.R., Vo A.Q., McFall H., Pimparade M.B., Bhagurkar A.M. (2018). Melt extrusion with poorly soluble drugs—An integrated review. Int. J. Pharm..

[3] Matić J., Paudel A., Bauer H., Garcia R.A.L., Biedrzycka K., Khinast J.G. (2020). Developing HME-Based Drug Products Using Emerging Science: A Fast-Track Roadmap from Concept to Clinical Batch. AAPS PharmSciTech.

[4] Park C., Renuka V.S., Lee B.J., de la Peña I., Park J.B. (2025). Advancements of hot-melt extrusion technology to address unmet patient needs and pharmaceutical quality aspects. J. Pharm. Investig..

[5] Melocchi A., Parietti F., Maroni A., Foppoli A., Gazzaniga A., Zema L. (2016). Hot-melt extruded filaments based on pharmaceutical grade polymers for 3D printing by fused deposition modeling. Int. J. Pharm..

[6] Korte C., Quodbach J. (2018). Formulation development and process analysis of drug-loaded filaments manufactured via hot-melt extrusion for 3D-printing of medicines. Pharm. Dev. Technol..

[7] Zhang J., Feng X., Patil H., Tiwari R.V., Repka M.A. (2017). Coupling 3D printing with hot-melt extrusion to produce controlled-release tablets. Int. J. Pharm..

[8] Bandari S., Nyavanandi D., Dumpa N., Repka M.A. (2021). Coupling hot melt extrusion and fused deposition modeling: Critical properties for successful performance. Adv. Drug Deliv. Rev..

[9] Dumpa N., Butreddy A., Wang H., Komanduri N., Bandari S., Repka M.A. (2021). 3D printing in personalized drug delivery: An overview of hot-melt extrusion-based fused deposition modeling. Int. J. Pharm..

[10] Arany P., Papp Á., Nemes D., Fehér P., Ujhelyi Z., Bácskay I. (2026). Quality by Design Approach for Hot-Melt Extrusion Coupled Fused Deposition Modeling (HME-FDM) 3D Printing: A Systematic Review. Pharmaceutics.

[11] Cailleaux S., Sanchez-Ballester N.M., Gueche Y.A., Bataille B., Soulairol I. (2021). Fused Deposition Modeling (FDM), the new asset for the production of tailored medicines. J. Control. Release Off. J. Control. Release Soc..

[12] Trenfield S.J., Awad A., Goyanes A., Gaisford S., Basit A.W. (2018). 3D Printing Pharmaceuticals: Drug Development to Frontline Care. Trends Pharmacol. Sci..

[13] Quodbach J., Bogdahn M., Breitkreutz J., Chamberlain R., Eggenreich K., Elia A.G., Gottschalk N., Gunkel-Grabole G., Hoffmann L., Kapote D. (2022). Quality of FDM 3D Printed Medicines for Pediatrics: Considerations for Formulation Development, Filament Extrusion, Printing Process and Printer Design. Ther. Innov. Regul. Sci..

[14] Mandrik M., Makarova V., Korol L., Krasnyuk I., Antonov S. (2026). Rational Design of Multicomponent Polymeric Systems Based on a Transient Plasticization Window for Hot-Melt Extrusion. Pharmaceutics.

[15] Ashokbhai M.K., Sanjay L.R., Sah S.K., Roy S., Kaity S. (2024). Premix technologies for drug delivery: Manufacturing, applications, and opportunities in regulatory filing. Drug Discov. Today.

[16] Butreddy A., Bandari S., Repka M.A. (2021). Quality-by-design in hot melt extrusion based amorphous solid dispersions: An industrial perspective on product development. Eur. J. Pharm. Sci..

[17] Kyeremateng S.O., Voges K., Dohrn S., Sobich E., Lander U., Weber S., Gessner D., Evans R.C., Degenhardt M. (2022). A Hot-Melt Extrusion Risk Assessment Classification System for Amorphous Solid Dispersion Formulation Development. Pharmaceutics.

[18] Uriel M., Marro D., Gómez Rincón C. (2023). An Adequate Pharmaceutical Quality System for Personalized Preparation. Pharmaceutics.

[19] Watson C.J., Whitledge J.D., Siani A.M., Burns M.M. (2021). Pharmaceutical Compounding: A History, Regulatory Overview, and Systematic Review of Compounding Errors. J. Med. Toxicol..

[20] U.S. Food and Drug Administration (2020). Draft Guidance for Industry. Current Good Manufacturing Practice—Guidance for Human Drug Compounding Outsourcing Facilities Under Section 503B of the FD&C Act: Guidance for Industry.

[21] National Association of Pharmacy Regulatory Authorities (2018). Guidance Document for Pharmacy Compounding of Non-Sterile Preparations.

[22] Secretan P.-H., Annereau M., Do B. (2025). A Risk-Based Framework for Hospital Compounding: Integrating Degradation Mechanisms and Predictive Toxicology. Pharmaceutics.

[23] Iyer R., Petrovska Jovanovska V., Berginc K., Jaklič M., Fabiani F., Harlacher C., Huzjak T., Sanchez-Felix M.V. (2021). Amorphous Solid Dispersions (ASDs): The Influence of Material Properties, Manufacturing Processes and Analytical Technologies in Drug Product Development. Pharmaceutics.

[24] Xie T., Taylor L.S. (2017). Effect of Temperature and Moisture on the Physical Stability of Binary and Ternary Amorphous Solid Dispersions of Celecoxib. J. Pharm. Sci..

[25] Lehmkemper K., Kyeremateng S.O., Heinzerling O., Degenhardt M., Sadowski G. (2017). Impact of Polymer Type and Relative Humidity on the Long-Term Physical Stability of Amorphous Solid Dispersions. Mol. Pharm..

[26] Moseson D.E., Taylor L.S. (2023). Crystallinity: A Complex Critical Quality Attribute of Amorphous Solid Dispersions. Mol. Pharm..

[27] Healy A.M., Worku Z.A., Kumar D., Madi A.M. (2017). Pharmaceutical solvates, hydrates and amorphous forms: A special emphasis on cocrystals. Adv. Drug Deliv. Rev..

[28] Grell T., Barbero M., Pattarino F., Giovenzana G.B., Colombo V. (2021). Solvatomorphism of Moxidectin. Molecules.

[29] Lehmkemper K., Kyeremateng S.O., Heinzerling O., Degenhardt M., Sadowski G. (2017). Long-Term Physical Stability of PVP- and PVPVA-Amorphous Solid Dispersions. Mol. Pharm..

[30] Theodosiou F., Blundell T.J., Evans J.S.O., Basford P., Fellah N., Cruz Cabeza A.J. (2026). Phase diagrams of pharmaceutical solvates from mechanochemistry. Nat. Commun..

[31] Klitou P., Pask C.M., Onoufriadi L., Rosbottom I., Simone E. (2020). Solid-State Characterization and Role of Solvent Molecules on the Crystal Structure, Packing, and Physiochemical Properties of Different Quercetin Solvates. Cryst. Growth Des..

[32] Zhang J., Guo M., Luo M., Cai T. (2023). Advances in the development of amorphous solid dispersions: The role of polymeric carriers. Asian J. Pharm. Sci..

[33] Jurczak E., Mazurek A.H., Szeleszczuk Ł., Pisklak D.M., Zielińska-Pisklak M. (2020). Pharmaceutical Hydrates Analysis—Overview of Methods and Recent Advances. Pharmaceutics.

[34] Yu K., Shen J., Liu J., Tang G., Hu X. (2023). Stability and Mechanical Properties of Darunavir Isostructural Solvates: An Experimental and Computational Study. Cryst. Growth Des..

[35] Kamat A.G., Ganala N.T., Boddu V.B., Koilpillai J.P., Meenaakshisunderam S. (2017). Solvates of Darunavir. U.S. Patent.

[36] Royal Society of Chemistry The Merck Index Online, Darunavir, Monograph M4099. https://merckindex.rsc.org/monographs/m4099.

[37] Smeets A., Koekoekx R., Clasen C., Van den Mooter G. (2018). Amorphous Solid Dispersions of Darunavir: Comparison Between Spray Drying and Electrospraying. Eur. J. Pharm. Biopharm..

[38] Modini A.K., Ranga M., Puppala U., Kaliyapermal M., Geereddy M.K.R., Samineni R., Grover P., Konidala S.K. (2023). Identification, Isolation, and Structural Characterization of Novel Forced Degradation Products of Darunavir Using Advanced Analytical Techniques Like UPLC-MS, Prep-HPLC, HRMS, NMR, and FT-IR Spectroscopy. Chromatographia.

[39] Thommes M., Baert L., Rosier J. (2011). 800 mg Darunavir Tablets Prepared by Hot Melt Extrusion. Pharm. Dev. Technol..

[40] Makarova V., Mandrik M., Antonov S. (2025). Laser Microinterferometry for API Solubility and Phase Equilibria: Darunavir as a Case Example. Pharmaceutics.

[41] Shi Q., Chen H., Wang Y., Wang R., Xu J., Zhang C. (2022). Amorphous Solid Dispersions: Role of the Polymer and Its Importance in Physical Stability and In Vitro Performance. Pharmaceutics.

[42] Shi Q., Li F., Yeh S., Wang Y., Xin J. (2020). Physical Stability of Amorphous Pharmaceutical Solids: Nucleation, Crystal Growth, Phase Separation and Effects of the Polymers. Int. J. Pharm..

[43] Mandrik M.A., Sadkovskii I.A., Pinegina E.D., Korol L.A., Krasnuk I.I. (2024). Development and Validation of UV-Spectrophotometry Method for Quantitative Determination of Amorphous Darunavir. Drug Dev. Regist..

[44] U.S. Food and Drug Administration Dissolution Methods Database.

[45] Corrêa J.C.R., Serra C.H.R., Salgado H.R.N. (2013). Stability Study of Darunavir Ethanolate Tablets Applying a New Stability-Indicating HPLC Method. Chromatogr. Res. Int..

[46] Winck J., Gottschalk T., Thommes M. (2023). Predicting Residence Time and Melt Temperature in Pharmaceutical Hot Melt Extrusion. Pharmaceutics.

[47] Ponsar H., Wiedey R., Quodbach J. (2020). Hot-Melt Extrusion Process Fluctuations and Their Impact on Critical Quality Attributes of Filaments and 3D-Printed Dosage Forms. Pharmaceutics.

[48] United States Pharmacopeial Convention (2019). General Chapter <701> Disintegration. USP–NF.

[49] United States Pharmacopeial Convention (2020). General Chapter <731> Loss on Drying. USP–NF.

[50] United States Pharmacopeial Convention (2022). General Chapter <905> Uniformity of Dosage Units. USP–NF.

[51] Jarrells T.W., Munson E.J. (2022). Comparison of Differential Scanning Calorimetry, Powder X-ray Diffraction, and Solid-state Nuclear Magnetic Resonance Spectroscopy for Measuring Crystallinity in Amorphous Solid Dispersions—Application to Drug-in-Polymer Solubility. J. Pharm. Sci..

[52] Merck KGaA (2022). Polyethylene Glycol 1500 for Synthesis, Product No. 807489; Product Specification/Safety Data Sheet.

[53] Tambe S., Jain D., Meruva S.K., Rongala G., Juluri A., Nihalani G., Mamidi H.K., Nukala P.K., Bolla P.K. (2022). Recent Advances in Amorphous Solid Dispersions: Preformulation, Formulation Strategies, Technological Advancements and Characterization. Pharmaceutics.

[54] Kawakami K., Ishitsuka T., Fukiage M., Nishida Y., Shirai T., Hirai Y., Hideshima T., Tanabe F., Shinoda K., Tamate R. (2025). Long-Term Physical Stability of Amorphous Solid Dispersions: Comparison of Detection Powers of Common Evaluation Methods for Spray-Dried and Hot-Melt Extruded Formulations. J. Pharm. Sci..

[55] S’ari M., Blade H., Cosgrove S.D., Drummond-Brydson R., Hondow N., Hughes L.P., Brown A. (2021). Characterization of Amorphous Solid Dispersions and Identification of Low Levels of Crystallinity by Transmission Electron Microscopy. Mol. Pharm..

[56] Purohit H.S., Zhang G.G.Z., Gao Y. (2023). Detecting Crystallinity in Amorphous Solid Dispersions Using Dissolution Testing: Considerations on Properties of Drug Substance, Drug Product, and Selection of Dissolution Media. J. Pharm. Sci..

[57] Antolović I., Vrabec J., Klajmon M. (2024). COSMOPharm: Drug–Polymer Compatibility of Pharmaceutical Amorphous Solid Dispersions from COSMO-SAC. Mol. Pharm..

[58] Bhatta H.P., Han H.-K., Maharjan R., Jeong S.H. (2025). Recent Techniques to Improve Amorphous Dispersion Performance with Quality Design, Physicochemical Monitoring, Molecular Simulation, and Machine Learning. Pharmaceutics.

[59] Jarrells T.W., Yuan X., Munson E.J. (2025). Impact of Storage Conditions on the Physical Stability of Amorphous Solid Dispersions Containing Two Structurally Similar Drugs. Mol. Pharm..

[60] Bahman M., Zini J., Lahtinen J., Hassinen N., Verma S., Laaksonen T., Airaksinen S., Sandler Topelius N., Viitala T. (2025). Assessment of a Semi-Solid Extrusion Based Compounding System Solution for Personalized Ondansetron Dosage Forms Combined with Raman Spectroscopy Analysis. Pharm. Res..

[61] Shokraneh F., Filppula A.M., Tornio A., Aruväli J., Paaver U., Sandler Topelius N. (2025). Automated Extrusion-Based Dispensing: Personalized Dosing and Quality Control of Clopidogrel Tablets for Pediatric Care. Eur. J. Pharm. Sci..

[62] Bernhardt M.B., Shokraneh F., Hrizanovska L., Lahtinen J., Brasher C.A., Sandler N. (2025). Automated 3D Printing-Based Non-Sterile Compounding Technology for Pediatric Corticosteroid Dosage Forms in a Health System Pharmacy Setting. Pharmaceutics.

[63] Ramos S., Vignes M., Secretan P.-H., Legrand F.-X., Annereau M., Do B. (2026). Hybrid GPP-Hospital Control Strategy for Semi-Solid Extrusion 3D Printing of ONC201 Chewable Units in Pediatric Oncology. Eur. J. Pharm. Sci..

